# Threatened species to super-abundance: The unexpected international implications of successful goose conservation

**DOI:** 10.1007/s13280-016-0878-2

**Published:** 2017-02-18

**Authors:** Anthony D. Fox, Jesper Madsen

**Affiliations:** Department of Bioscience, Aarhus University, Kalø, Grenåvej 14, 8410 Rønde, Denmark

**Keywords:** Geese and agriculture, Goose hunting, Over exploitation, Population growth, Protection, Refuge

## Abstract

Wild geese wintering in western Europe were declining by the 1930s probably due to loss of natural habitat and over exploitation through hunting, although the causes will never be known. Refuge provision and hunting restrictions from the 1950s enabled numbers to recover. Improved monitoring systems enabled the description of progressive increases and extensions of wintering range since that time, especially amongst those goose populations that increasingly exploited agricultural landscapes. This introductory article sets the scene for the special issue on the increasing interactions and conflicts created by recent increases in the range and abundance of wild geese throughout the northern hemisphere, especially with regard to agricultural damage, but including issues associated with air flight safety, human and animal health, ecosystem effects and conflicts with other biodiversity objectives. It also provides the context for finding common solutions to problems, presenting experiences from regional-, national- and flyway-coordinated management to find solutions to conflict.

## Introduction: the awakening of western European conservation awareness

When one reads the heroic tales of the mass slaughter of geese by the great British wildfowlers of the nineteenth century, one could be forgiven for thinking that the 1800s were a period of enormous goose abundance in the United Kingdom, characterised by skies black with geese and unimaginable daily bags (e.g. Folkard 1859; Hawker 1893; Chapman 1928). By comparison, much later, seen from the standpoint of western European observers in the 1930s, there seemed good reason to believe that there had been catastrophic declines in many wildfowl species, including geese, during the first half of the last century. This belief gave rise to the instigation of an International Wildfowl Inquiry into the European status of ducks and geese (Berry 1939a, b) by the British Section of the International Committee for Bird Preservation (the precursor of BirdLife International).

The evidence gathered by the Inquiry suggested that goose populations had been, and at that time continued to be, threatened by over exploitation through the improvement and accessibility of firearms, the economic development of much marginal and wetland habitat across Europe, and what was then also considered to be unsustainable exploitation on breeding areas [much later revealed by Storå (1968) and Nowak (1995)]. This led the Inquiry to conclude that there was a radical need to instigate protection of geese and habitats to restore them to what we might now call favourable conservation status. There was no doubt that even before the Second World War, some goose populations were in trouble. For some species, this was the result of long-term effects of persecution, as was the case for the greylag goose *Anser anser* which was extirpated as a breeding bird throughout much of England in the late 1700s and had become restricted to a few Hebridean breeding refugia by the 1880s (Holloway 1996). Wintering numbers of various goose species in the Rhine-Meuse Delta (a current stronghold for several species) were considered “on the brink of extinction” by the 1930s (Neinhuis 2008). Furthermore, the “wasting disease” of *Zostera* associated with the mycetozoan *Labyrinthula* in the Northern Hemisphere that affected extensive areas of this plant in 1931 and 1932 had a profound effect on the brent goose *Branta bernicla* populations that almost exclusively relied on this winter food source (Cottam et al. 1944; Cottam and Munro 1954; Rasmussen 1977), although it has been argued that exploitation may have controlled the remaining small populations (Madsen 1987; Ebbinge 1991). On a continent already ravaged by the Second World War, post war reconstruction went on to contribute to the destruction and degradation of wetlands and natural goose habitat across Europe. Poorly or unrestricted regulation of hunting (often commercially motivated) by a hungry populace further adversely impacted populations. In the 1950s, there were very few protected areas for any form of wildlife and even as rudimentary site-safeguard mechanisms began to emerge, for geese these were typically only night time roosts leaving them vulnerable to wildfowling during their feeding flights elsewhere. Goose population structure, flyways, status, abundance, trends and distribution were still poorly known.

## Changing attitudes and restrictive hunting legislation: the case of the United Kingdom

All this began to change radically at the beginning of the 1950s. Knowledge of flyways improved through the capture and individual marking of geese with metal rings thanks to pioneers such as Sir Peter Scott’s Severn Wildfowl Trust (e.g. Scott et al. 1953). These generated patterns of ringing recoveries that enabled definition of discrete flyway populations that used separate breeding, moulting, staging and wintering areas (e.g. Boyd and Scott 1955). Knowledge of these relationships enabled coordinated international counts to assess discrete population sizes and to start annual surveillance which, over time, generated knowledge about changes in abundance (e.g. Boyd 1961). These pioneering attempts at census generally painted a picture of slowly increasing numbers, but concern still focussed upon the fact that in the 1950s, many of these populations were thought to be showing the first signs of recovery from very low population levels. This meant that conservation actions were necessary to support their continued growth, primarily through regulation of hunting pressure prevailing at the time.

Spring shooting of geese had been made unlawful in the United Kingdom as long ago as 1881, but hunting generally continued to adversely impact upon populations between the World Wars. In the UK, the Duck and Goose Act of 1939 protected wildfowl from hunting between 1 February and 11 August and the 1954 Protection of Birds Act outlawed large barrelled guns above 4.5-cm diameter and removed brent and barnacle geese *Branta leucopsis* entirely from the hunting list. The legislation was strengthened in 1967 by prohibiting the marketing of shot geese, which was the final piece of legislation making a substantial difference to the level of shooting mortality across all wild goose species in the United Kingdom (although single species legislation followed with the 1981 Wildlife and Countryside Act).

### Development of protected area networks in the United Kingdom

As well as reducing hunting mortality, it was also recognised that much of the habitat used by geese was under threat from development pressures. The few protected areas that existed prior to the 1950s in the UK were greatly limited in extent and distribution, and typically restricted to roost areas only. At that time, the Nature Conservancy UK established a Wildfowl Conservation Committee, who recognised “…that an adequate and suitably administered series of wildfowl refuges form a desirable and, in some conditions, an indispensable means of conserving and increasing wildfowl stocks, in which wildfowlers are no less interested than protectionists and scientists” (Wildfowl Conservation Committee 1961). National Wildfowl Refuges were established from 1955 onwards on the Humber Estuary, at Southport on the Ribble Estuary and Caerlaverock on the Solway Firth in Scotland and were complemented by networks of National Nature Reserves and Sites of Special Scientific Interest which enabled site protection under the provisions of the National Parks and Access to the Countryside Act 1949 (see Ratcliffe 1977; Poore and Gryn-Ambroes 1980). Nonetheless even in the 1960s, there were no effective international frameworks within which to start to develop cohesive site-safeguard networks or coordinated approaches to hunting exploitation other than at national scales, while overall knowledge of numbers and trends remained extremely poor.

## Results of recent population monitoring

Knowledge of discrete population flyways and abundance are now essential foundations for the constructions of site-safeguard networks, so, for instance, the designation of key sites supporting more than 1% of goose flyway populations underpins the UK’s commitments under contemporary legislation such as designating Wetlands of International Importance under the Ramsar Convention and contributes to designation of Special Protection Areas under the EU Bird’s Directive (Stroud et al. 2001, 2016).

**Fig. 1:**
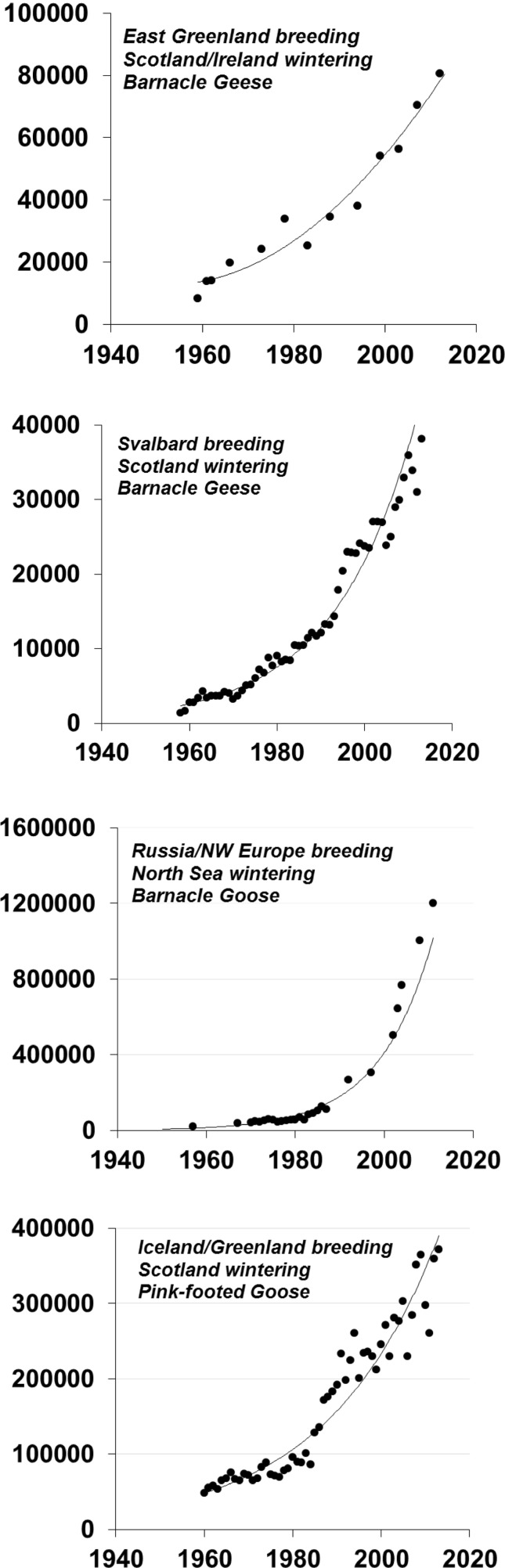
Examples of four populations of western European goose populations showing current exponential growth (from Fox and Leafloor unpublished results)

**Fig. 2:**
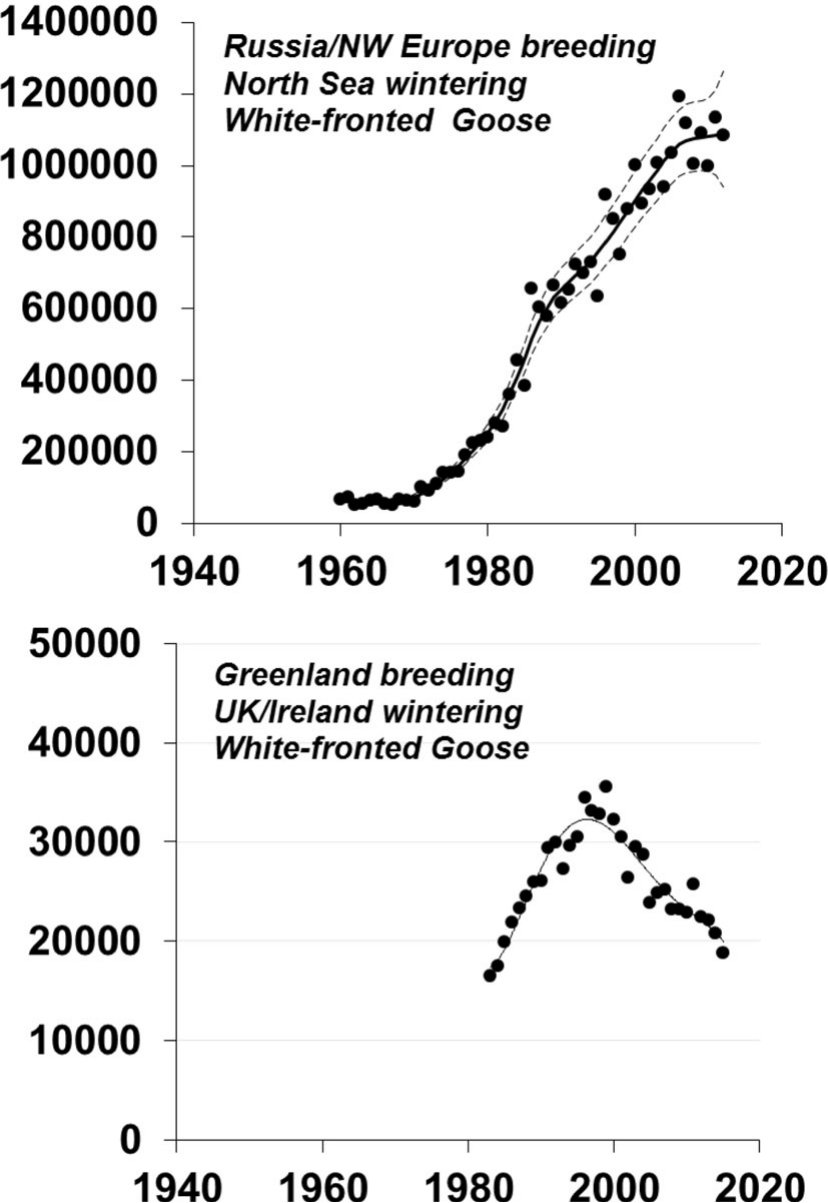
Examples of two populations of western European goose populations showing stabilisation (*upper Anser albifrons albifrons* showing modelled 95% confidence intervals) or decline (*lower A.a. flavirostris*) in growth rates (from Fox and Leafloor unpublished results)

In the face of our still relatively flawed modern monitoring programmes, it is important to remember the limitations of historical goose abundance data for drawing any significant inference about genuine changes in abundance. The recent review of the status and abundance of 69 populations of 15 species of northern hemisphere geese found that less than half of all current estimates of population size were likely to fall within 10% of the true number, and most of the best estimates were from North American populations (Fox and Leafloor unpublished results). None of the time series that exist extend before the early 1950s. For this reason, it is extraordinarily difficult to assess the population size of many goose populations before the middle of the last century. Despite this lack of historical context, our current knowledge has been invaluable for establishing the general abundance and recent trends in most western European goose populations. Furthermore, marking programmes throughout the latter half of the last century has contributed enormously to improve our understanding of flyway population definition. All these monitoring programmes show that numbers of geese of the majority, but not all, of western European goose populations have increased dramatically in Europe since 1960s (e.g. Fox et al. 2010; Fox and Leafloor unpublished results). Many of these populations show unchecked exponential increase since systematic counting began (Fig. 1), although a few show stabilisation and recent declines (Fig. 2). Of 17 populations with known longer-term trends in western Europe, 14 are currently showing significant exponential increases and only three declining (Table 1). The seven goose populations in the United Kingdom that summed to 100 000 birds in the 1950s now number over a million individuals (Mitchell et al. 2010).

**Table 1:** The most recent estimated population sizes of 17 wild goose populations in western Europe as reviewed in Fox and Leafloor (unpublished results). Columns also provide the year of the estimate, as well as the longer-term (>10 years but time series depending on population) trends expressed as percentage rate of change per annum, together with the duration of the period use to calculate these trends

Goose species and population	Estimated population size	Year of estimate	% rate of change per annum	Period of rate of change
Taiga Bean Goose *Anser fabalis fabalis*	52 000	2015	−6.0	2006–2015
Tundra Bean Goose *Anser fabalis rossicus*	600 000	2014	+2.6	1990–2013
Iceland Pink-footed Goose *Anser brachyrhynchus*	360 000	2013	+3.9	1960–2013
Svalbard Pink-footed Goose *Anser brachyrhynchus*	76 000	2014	+3.6	1965–2013
Baltic-North Sea wintering White-fronted Goose *Anser albifrons albifrons*	1 085 000	2012	+2.5	1988–2012
Greenland White-fronted Goose *Anser albifrons flavirostris*	18 900	2015	−2.8	1999–2014
Scandinavian Lesser White-fronted Goose *Anser erythropus*	80	2010	−5.0	1993–2008
Iceland Greylag Goose *Anser anser*	100 000	2014	+1.5	1960–2013
UK breeding Greylag Goose *Anser anser*	140 000	2014	+9.4	1998–2008
NW Europe breeding Greylag Goose *Anser anser*	960 000	2014	+8.5	1980–2008
Central European Greylag Goose *Anser anser*	100 000	2014	+6.8	1995–2008
Greenland Barnacle Goose *Branta leucopsis*	80 500	2013	+3.6	1959–2012
Svalbard Barnacle Goose *Branta leucopsis*	38 000	2013	+6.6	1956–2013
Russia/Baltic/North Sea Barnacle Goose *Branta leucopsis*	1 200 000	2015	+7.8	1960–2014
Russian Dark-bellied Brent Goose *Branta bernicla bernicla*	211 000	2011	+5.6	1956–2010
NE Canada light-bellied Brent Goose *Branta bernicla hrota*	48 000	2011	+4.4	1996–2013
Svalbard light-bellied Brent Goose *Branta bernicla hrota*	7500	2015	+2.4	1987–2015

## Changes in habitat use: the switch from natural to agricultural foods

While it is tempting to suggest that reductions in hunting mortality and the designation of protected areas supported the expansion in numbers of geese in western Europe, we have no data on the specific effects of these actions on goose demography to support these hypotheses from that time. Furthermore, it is clear from the counts in very recent years that exponential increases in numbers of many goose populations continue (e.g. Fig 1; Table 1) and many populations that specifically exploit agricultural habitats show expansions in wintering range.

A feature associated with many increases in goose population size has been the shift in their habitat utilisation from natural wetlands to temperate farmland landscapes where they have become adept at exploiting agricultural crops and all forms of managed grassland (e.g. Abraham et al. 2005; Fox et al. 2005; Fox and Abraham 2017). This would suggest that temperate agriculture has been highly effective at extending the effective carrying capacity of wintering goose numbers (van Eerden et al. 1996). As large herbivorous birds with a relatively simple gut structure, geese have traditionally aggregated to natural plant communities that offer a dense source of food (Fox et al. 2017). For instance, several short-billed goose species traditionally grazed the above ground biomass of short sward, graminoid-dominated low diversity plant communities. Such communities can be found in the intertidal (where geese graze *Zostera*) and subtidal (where geese up-end to browse submerged *Zostera*) in the case of many populations of brent geese on salt stressed grasslands, such as saltmarshes (e.g. Svalbard and Greenland barnacle geese and dark-bellied brent geese *B. b. bernicla*) and clifftop and dune grassland (Greenland barnacle geese). Now, the same goose species find such highly nutritious short swards in agricultural and other artificial landscapes, such as in intensively managed pasture, amenity grasslands and winter cereal fields. Other goose species with more robust bills and necks combine grazing of longer, coarser swards [e.g. western taiga bean geese *Anser fabalis fabalis* (Allport 1991)] with digging in wet substrates to extract the below ground overwinter perenniating parts of plants [such as *Scirpus* species in saltmarshes in the case of greylag and snow geese *Chen caerulescens* (Amat 1995), and *Eriophorum angustifolium* in surface patterned mires in the case of the Greenland white-fronted geese *A. albifrons flavirostris* (Ruttledge 1929; Fox et al. 1990)]. These goose species now find such food in the form of agricultural products, such a root crops (e.g. potatoes and beet) and grain (especially cereal and maize left after the harvest).

In western Europe, intensification of agriculture has increasingly made farmland landscapes homogeneous, stimulated by technological change and the EU’s Common Agricultural Policy. Although St. Werburgh was banishing geese from English fields in the seventh century (Kear 2001), until the last 70 years, agriculture has never served up such rich monocultures of goose food as is the case in contemporary Europe. Serried ranks of sown rows of single species of grass and arable crops, waste root crops, unharvested and spilled cereal grains (all selectively bred for their food quality) provide monocultures of high quality food for geese. Such resources support unimaginably high food intake rates compared to those possible when foraging on saltmarshes or even low intensity pastures, where birds are constrained to search amongst diverse swards for the most nutritionally rewarding grass blades or other sources of wild foods (e.g. Madsen 1985; Therkildsen and Madsen 2000; Fox et al. 2005; Fox and Abraham 2017). Little wonder that, in response, geese have progressively abandoned their natural habitats to exploit this larder of super-abundance and, when scared away, show little desire to abandon nutritionally rich agricultural fields for the natural and semi-natural habitats that were exclusively their former natural foraging habitats.

However, in making these transitions, these patterns have created a series of problems and challenges to a range of stakeholders and government agencies for the effective management of goose populations. For example, Chapman (1928) described brent geese as never touching “…British soil, being exclusively marine…they sleep at sea and only enter tidal mud-flats to feed…never go inland, nor trespass a single yard above the full sea-mark”. Yet by the early 1980s the species had begun to feed on pasture, winter cereal and oil seed rape over the seawall from their former saltmarsh and *Zostera* beds in southern Britain to the extent that they had become a cause of major loss of income to farmers and a major locus for conflict (e.g. Vickery and Summers 1992; McKay et al. 1993, 1994, 1996a, b; Vickery et al. 1994). In Ireland, brent geese now regularly feed amongst dog walkers and football players in Dublin parks.

There is little convincing evidence that the move by geese from feeding on natural or semi-natural habitats to completely artificial ecosystems has had impacts at the population level. The only study to establish a link between demographical parameters and the shifts to agricultural feeding comes from the study of winter site-faithful Greenland white-fronted geese, which showed flocks wintering on intensively managed agricultural land produced 10% more young than those that remained feeding exclusively on natural peat bog vegetation (which were numerically far smaller and therefore contributed very much fewer young to future generations than did farmland flocks, Fox et al. 2005).

Despite our inability to directly link the switch from natural wetlands to farmland with increases in population size, support for this hypothesis comes from the East Asian flyway populations of geese. In China, human persecution on farmland means that wintering wild goose populations remain almost entirely dependent on wetlands for feeding in winter. Most wintering goose species are declining in China because of the hydrological and trophic changes in wetlands that are increasingly denying them of food (e.g. Fox et al. 2011; Zhao et al. 2012; Wang et al. 2013). The exception is the eastern tundra bean goose, *Anser fabalis serrirostris*, which does feed on farmland and which seems to be maintaining stable or increasing numbers (Zhao et al. 2010; Jia et al. 2016). Furthermore, some of the same goose species that are declining in China feed on spilled wheat and rice grains in Korea and Japan where their wintering numbers are showing similar increases to continental North America and Europe. This demonstrates that the same goose species within the same flyway thrive on farmland in situations where they are free to exploit such sources of food (Jia et al. 2016) and supports the hypothesis that the shift to farmland has contributed to increases in abundance.

While there is only circumstantial evidence that the shift from natural habitats to agricultural ones has fuelled the rapid increase to specific goose populations in recent years, the fact remains that now these herbivorous birds have learnt to exploit such landscapes, the modern farmland of their wintering range offers currently unlimited access to food during the non-breeding periods of the annual cycle, which means that winter forage in the immediate future is not likely to be a limiting factor. That said, the increasing reliance on agricultural landscapes of goose populations in Europe and North America does make them dependent upon current patterns of cropping and agriculture. This puts goose populations at the mercy of major changes to the farming landscape patterns brought about by globalisation, politics, climate change and farming developments which may conspire to drive agricultural change in unpredictable ways that will not necessarily be beneficial for geese in the future (Fox and Abraham 2017).

Since the exponential increases in most common goose populations show little sign of stabilising (however, see Fig. 2), this may also be the case for the breeding areas as well. Some studies show that increasing goose populations wintering in temperate regions may have major local impacts on Arctic ecosystems in the form of eutrophication and reduction of vegetation cover as a result of grazing (Madsen et al. 2011; Hassen et al. 2016). Hence, increases in goose abundance may have longer-term negative consequences for the number of geese the habitat can sustain. In general, however, we see few signs of strong density dependence at the population level that might limit the growth rate of these populations in the near future.

## Finding solutions to conflicts caused by increasing goose abundance: an introduction to articles in this special issue

As numbers of wintering geese have increased in Europe, so the degree and geographical extent of conflict with farming interests has increased as a recent review has shown (Fox et al. 2017). However, it has become increasingly apparent that despite the primary focus in the agricultural arena, issues associated with air flight safety, human and animal health, ecosystem effects and conflict with other biodiversity objectives have also been rising up the political agenda.

These multiple societal challenges require careful integration for their successful resolution, and the aim of this special issue of *Ambio* is to bring together some of the most experienced professionals in their fields to review the strengths and weaknesses of existing attempts to integrate these multiple challenges in cohesive goose management programmes. In particular, effective mechanisms for integrating diverse and conflicting interests, using interdisciplinary approaches at local, regional, national and flyway scales are sought, incorporating participatory and adaptive approaches. Lefebvre et al. (2017) and Madsen et al. (2017) review some of the fundamentals of what causes conflict in relation to specific populations of wild geese and some of the mechanisms for deconstructing and finding solutions to such conflicts. Inevitably, there is considerable need for emphasis upon understanding the nature of the conflict between geese and agriculture. Simonsen et al. (2017) consider scaring as a tool to alleviate crop damage by geese, but also look at how farmers perceive goose damage. They show that the degree of scaring effort invested by a farmer is not necessarily a direct function of goose use of his farm, underlining the need to better understand the sociological factors that shape perceptions in these and other such conflicts. Assessing effectiveness of regional management is undertaken specifically with respect to agriculture in Norway by Baveco et al. (2017), complemented by a review of the success and value of key approaches to resolving conflict on the Scottish island of Islay (McKenzie and Shaw 2017). Islay experiences particular problems because of the internationally important concentrations of goose populations of conservation importance which occur on the island and contribute to the green economy there, but that nevertheless cause conflict with farmer’s incomes (McKenzie and Shaw 2017). The Islay case study is also set in the context of examining how regional management fits within the context of a national strategy and how it compares with other goose management schemes throughout Scotland (Bainbridge 2017). We also try to understand the strengths and weaknesses of national approaches that have been tried and tested in Norway (Eythórsson et al. 2017), the Netherlands (Koffijberg et al. 2017) and how effective interventions against burgeoning numbers of breeding geese are being dealt with in the Netherlands as this important issue begins to rise up the agendas of western European governments (van der Jeugd and Kwak 2017). Overabundant geese populations have been a problem recognised for a rather longer time period in North America than in Europe. For this reason, we also review how American plans for managing goose populations have progressed, delivered and developed with particular emphasis on delivering key recommendations about pitfalls to avoid as well as concentrating on highlighting the best mechanisms for delivery (e.g. Lefebvre et al. 2017). Many of the experiences associated with adaptive harvest management gained in North America have been applied to a pioneering process applied to the Svalbard-breeding population of the pink-footed goose and the knowledge gained at every step in the development of this unique European management system is presented in Madsen et al. (2017). It is also becoming abundantly evident that changes in goose abundance are having considerable societal and ecological impacts away from commercial damage to agricultural interests, so we summarise available experiences arising from the increase in air flight safety issues related to geese associated with airports around the world (Bradbeer et al. 2017) as well as reviewing the knock-on effects of goose distribution and abundance on ecosystems and other organisms in general (Buij et al. 2017). Finally, we round off with a summary and synthesis of the entire exercise where specific recommendations are made to take the process forward (Stroud et al. 2017). There was clearly a very pressing need for such a synthesis and we are confident that we have been able to gather a unique set of experiences from practitioners around the globe from which to distill the most effective mechanisms available to form the basis for taking forward ideas about how to mount a successful integrated, multi-layered approach to goose management at a strategic level in the future.
